# Supplementary material for: Early Supplementation with Branched-Chain Amino Acids Ameliorates Lipid Retention in Aortic Valves of ApoE-Knockout Mice
Source: Int J Mol Sci. 2025 Nov 21;26(23):11259. doi: 10.3390/ijms262311259 (PMC12692212; doi:10.3390/ijms262311259)
Supplement: Supplementary file 1 [file ijms-26-11259-s001.zip › ijms-3957153-supplementary.docx]

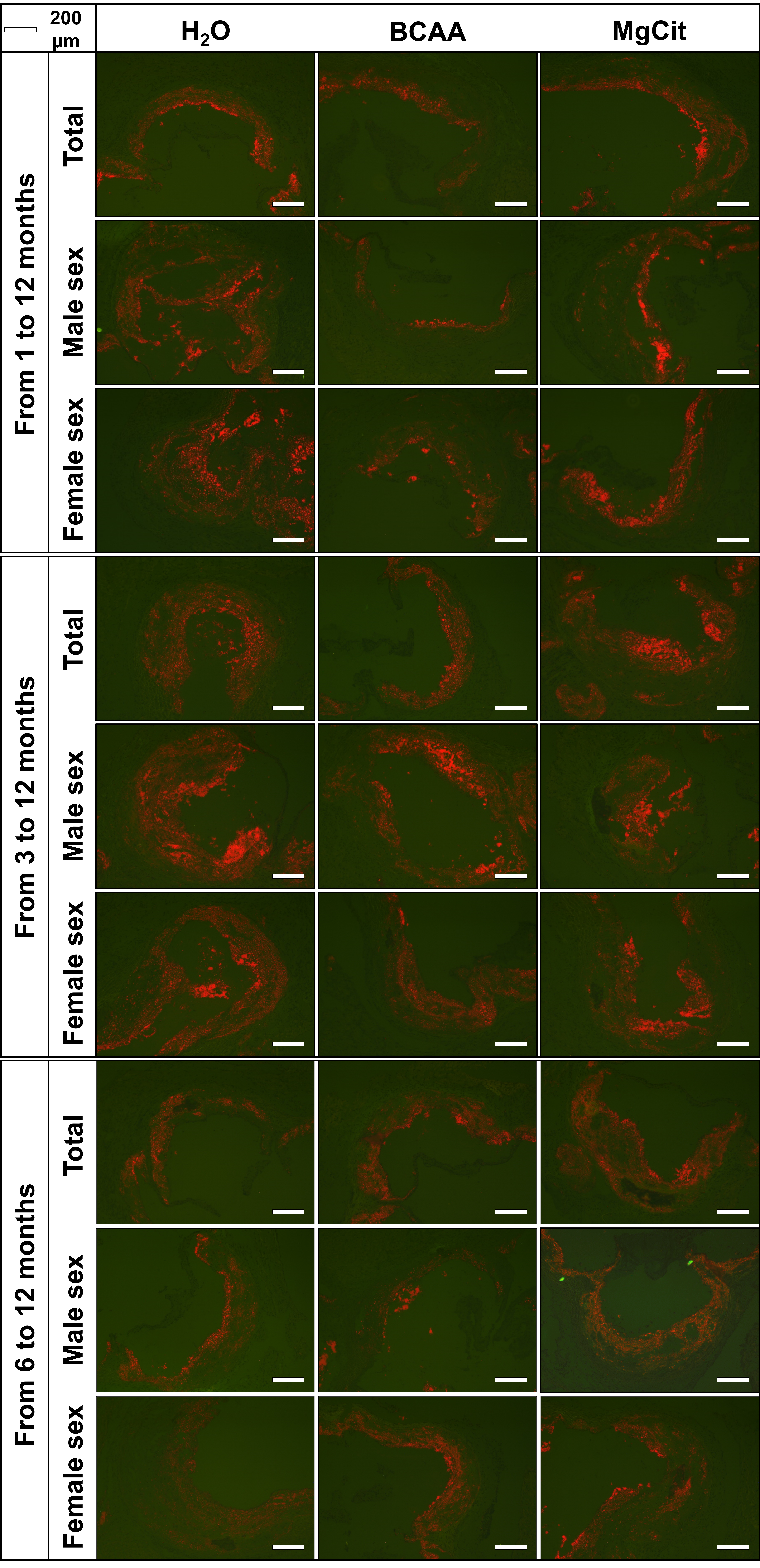


**Figure S1.** Aortic valves of ApoE-knockout mice treated with BCAA (55 mg/day, which corresponds to a human equivalent dose of 13.5 g/day) or MgCit (1.85 mg/day, which corresponds to a human equivalent dose of 450 mg/day) from 1 to 12 months of age (11-month treatment duration, top), from 3 to 12 months of age (9-month treatment duration, center), or from 6 to 12 months of age (6-month treatment duration, bottom). Mock-treated mice received water without any supplements. Representative fluorescence microscopy images of Oil Red O-stained aortic valve sections. Scale bar: 200 µm.


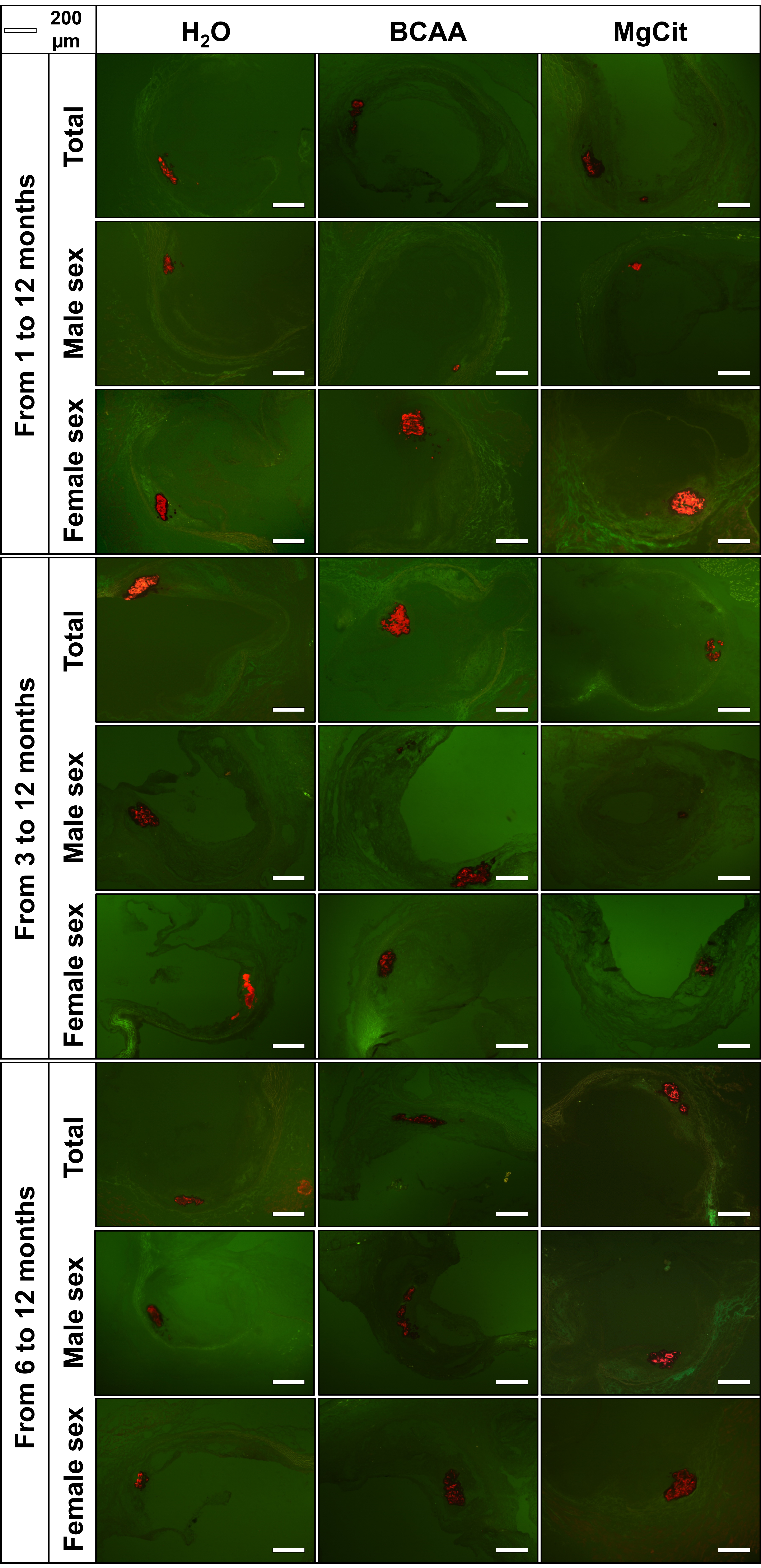


**Figure S2.** Aortic valves of ApoE-knockout mice treated with BCAA (55 mg/day, which corresponds to a human equivalent dose of 13.5 g/day) or MgCit (1.85 mg/day, which corresponds to a human equivalent dose of 450 mg/day) from 1 to 12 months of age (11-month treatment duration, top), from 3 to 12 months of age (9-month treatment duration, center), or from 6 to 12 months of age (6-month treatment duration, bottom). Mock-treated mice received water without any supplements. Representative fluorescence microscopy images of Alizarin Red S-stained aortic valve sections. Scale bar: 200 µm.
